# IGCCCG-Fehlklassifikation (International Germ Cell Consensus Classification) durch zeitlich inkorrekte Interpretation der Serumkonzentration der Tumormarker bei metastasierten testikulären Keimzelltumoren

**DOI:** 10.1007/s00120-020-01432-1

**Published:** 2021-01-11

**Authors:** Pia Paffenholz, Tim Nestler, Yasmine Maatoug, Melanie von Brandenstein, Barbara Köditz, David Pfister, Axel Heidenreich

**Affiliations:** 1 Klinik für Urologie, Uro-Onkologie, spezielle urologische und roboter-assistierte Chirurgie, Universitätsklinikum Köln, Kerpener Str. 62, 50937 Köln, Deutschland; 2 Department of Urology, Federal Armed Services Hospital Koblenz, Koblenz, Deutschland; 3 Department of Urology, Medical University Vienna, Vienna, Österreich

**Keywords:** IGCCCG-Klassifikation, Testikuläre Keimzelltumoren, Tumormarkerserumkonzentrationen, Ablatio testis, Chemotherapie, IGCCCG, Testicular germ cell tumors, Tumor marker serum concentration, Orchiectomy, Chemotherapy

## Abstract

**Hintergrund:**

Das Klassifikationssystem zur Prognoseeinschätzung der International Germ Cell Cancer Cooperative Group (IGCCCG) für testikuläre Keimzelltumoren basiert auf dem histologischen Subtyp, der Lokalisation des Primärtumors und der Metastasen sowie der Serumkonzentrationen der Tumormarker vor Chemotherapie.

**Fragestellung:**

Ziel der Arbeit war die Evaluation des Einflusses der Verwendung der Tumormarkerserumkonzentrationen vor Ablatio testis im Vergleich zu denen vor Chemotherapie im Hinblick auf die Eingruppierung entsprechend der IGCCCG-Klassifikation.

**Material und Methoden:**

Wir führen eine retrospektive Datenanalyse an 135 Patienten mit metastasiertem testikulärem Keimzelltumor durch, die eine Primärtherapie mit einer Chemotherapie erhalten haben. Es erfolgte die Analyse von klinischen Parametern mit Fokus auf der Tumormarkerserumkonzentration vor Ablatio testis und vor Chemotherapie, die zur Eingruppierung in eine Prognosegruppe entsprechend der IGCCCG-Klassifikation führten.

**Ergebnisse:**

Die Verwendung der Tumormarkerserumkonzentrationen zur Berechnung der IGCCCG-Klassifikation vor der Ablatio testis im Vergleich zu denen vor Chemotherapie führte bei 8 % (11/135) aller Patienten zu einer veränderten Prognosegruppe sowie daraus folgend nicht-leitliniengerechten Therapieschemata. Es zeigt sich ein „up-staging“ bei 8 der 11 Patienten und somit 6 % (8/135) der gesamten Patientenkohorte, d. h. die Serumkonzentrationen der Tumormarker sind nach Ablatio bis zum Beginn der Chemotherapie abgefallen. Bei 3 der 11 Patienten bzw. 2 % (3/135) der gesamten Patientenkohorte, kam es zu einem „down-staging“, d. h. die Tumormarker sind bis zum Beginn der Chemotherapie angestiegen.

**Diskussion:**

Die Verwendung der Tumormarkerserumkonzentrationen vor Ablatio testis im Vergleich zu denen vor Chemotherapie kann zu einer signifikanten IGCCCG-Fehlklassifikation und somit inkorrekter Therapie führen. Für ein leitlinienkonformes „staging“ der Patienten sollten folglich die Tumormarker vor der Chemotherapie verwendet werden.

## Abstract (en)

**Background:**

The prognostic classification system of the International Germ Cell Cancer Cooperative Group (IGCCCG) for testicular germ cell tumors is based on the histological subtype, location of the primary tumor, extent of metastatic spread and prechemotherapy tumor marker serum concentrations.

**Objectives:**

In this study, we aim to identify whether the use of preorchiectomy instead of prechemotherapy tumor marker serum concentration has an impact on IGCCCG risk group assignment.

**Materials and methods:**

We performed a retrospective analysis including 135 patients with metastasized testicular germ cell tumors. Analysis of the clinical information with a focus on the tumor marker serum concentration preorchiectomy and prechemotherapy was performed, thus leading to the grouping of patients according to IGCCCG risk group assignment.

**Results:**

Using preorchiectomy instead of prechemotherapy tumor markers led to an incorrect IGCCCG risk group classification in 8% (11/135) of all patients, and consequently to a non-guideline concordant treatment. Up-staging was observed in 8 of 11 patients, representing 6% (8/135) of the total patient cohort. Three of the 11 misclassified patients showed a down-staging and thus describe 2% (3/135) of the total patient cohort.

**Conclusions:**

Using preorchiectomy tumor markers instead of prechemotherapy serum concentration might lead to an incorrect IGCCCG risk group assignment as well as non-guideline concordant treatment. Consequently, prechemotherapy tumor marker serum concentration should be applied for guideline concordant staging of patients.

Das Klassifikationssystem der International Germ Cell Cancer Cooperative Group (IGCCCG) zur Prognoseeinschätzung der testikulären Keimzelltumoren basiert neben dem histologischen Subtyp, der Lokalisation des Primärtumors und Metastasen v. a. auf den Tumormarkerserumkonzentrationen vor Chemotherapie. Eine vorherige Studie zeigte eine signifikante IGCCCG-Fehlklassifikation bei Verwendung der Tumormarker vor Ablatio testis anstelle derer vor Chemotherapie. Die aktuelle Analyse beschäftigt sich mit der klinischen Relevanz dieser Fehlklassifikation sowie dem Ausmaß einer potenziellen Unter- bzw. Übertherapie.

## Hintergrund und Fragestellung

Entsprechend der aktuellen Leitlinienempfehlung werden metastasierte testikuläre Keimzelltumor anhand des Klassifikationssystems der IGCCCG in entsprechende Prognosegruppen eingeteilt, nach der die Intensität der weiteren Therapie festgelegt wird (Tab. 1; [8, 15]). Dabei werden sowohl der histologische Subtyp, die Lokalisation des Primärtumors sowie der Metastasen als auch die Serumkonzentrationen der Tumormarker Alpha-Fetoprotein (AFP), humanes Choriogonadotropin (HCG) und Laktatdehydrogenase (LDH) berücksichtigt [1]. Da die Tumormarker nach erfolgter Ablatio testis üblicherweise abfallen, werden hier die Tumormarkerserumkonzentrationen vor Chemotherapie verwendet, die somit die Metastasenlast beschreiben [1]. Entsprechend der aktuell gültigen Leitlinien sollten die Tumormarker für eine korrekte S‑Klassifikation an Tag 1 des ersten Chemotherapiezyklus bestimmt werden [1]. Werden hingegen fälschlicherweise präoperative Tumormarker verwendet, besteht die Gefahr einer falsch-hohen Risikoklassifizierung und daraus resultierenden Übertherapie [5]. Die Folgen der Übertherapie sind chemotherapieassoziierte Akut- und Langzeittoxizitäten mit dosiskumulativen kardiovaskulären, neurologischen, nephrologischen oder endokrinologischen Auswirkungen [2–4, 6, 7, 9, 10]. In selteneren Fällen kommt es postoperativ zu einem Anstieg der Hodentumormarker, so dass auch eine Unterklassifizierung mit der Gefahr einer Untertherapie dieser Patienten möglich ist. Es konnte bereits anhand einer monozentrischen Studie mit kleiner Fallzahl von 83 Patienten gezeigt werden, dass es bei der Nutzung von präoperativen Tumormarkern zu einer Fehlklassifikation der IGCCCG-Risikogruppe kommen kann [5].

**Table 1:** 

Klassifikation entsprechend der IGCCCG	
**Gute Prognose**	
*Seminom (90* *% aller Fälle)*	*Nicht-Seminom (56* *% aller Fälle)*
Jede Primärlokalisation	Gonadaler/retroperitonealer Primärtumor
Keine extrapulmonalen Metastasen	Keine extrapulmonalen Metastasen
Jede Markerkonstellation	S0–S1
**Intermediäre Prognose**	
*Seminom (10* *% aller Fälle)*	*Nicht-Seminom (28* *% aller Fälle)*
Jede Primärlokalisation	Gonadaler/retroperitonealer Primärtumor
Extrapulmonalen Metastasen	Keine extrapulmonalen Metastasen
Jede Markerkonstellation	S2
**Schlechte Prognose**	
*Seminom – nicht klassifiziert*	*Nicht-Seminom (16* *% aller Fälle)*
	Mediastinaler Primarius
	Extrapulmonale Metastasen
	S3

*IGCCCG* International Germ Cell Cancer Collaborative Group

Das Ziel dieser Studie war die Evaluation der klinischen Relevanz dieser Fehlklassifikation entsprechend der IGCCCG-Prognosegruppen durch die Verwendung von präoperativen Tumormarkern mit Beschreibung des Ausmaßes einer potenziellen Unter- bzw. Übertherapie.

## Methoden

### Allgemeines

Wir führte eine retrospektive Datenanalyse an Patienten mit metastasiertem testikulärem Keimzelltumor (ICD-10 Code C62) durch. Von den 810 Patienten, die zwischen 2010 und 2020 aufgrund ihrer Erstdiagnose oder für Folgeuntersuchungen in unserer Klinik für Urologie therapiert wurden, wiesen 356 ein metastasiertes Stadium auf. Von diesen konnten 135 Patienten mit einem kompletten Datensatz inkludiert werden. Patienten mit fehlenden Daten (wie z. B. fehlende Tumormarker) wurden exkludiert. Wir analysierten klinische Parameter der Patienten mit Fokus auf den Tumormarkerserumkonzentrationen vor Ablatio testis und vor Chemotherapie, die zur Eingruppierung in eine Prognosegruppe entsprechend der IGCCCG-Klassifikation führten (Tab. 1; [8, 15]). Die IGCCCG-Risikogruppenzuweisung wurde korrekt durchgeführt mit den Tumormarkerserumkonzentrationen vor der Chemotherapie und verglichen mit den inkorrekt durchgeführten Klassifikationen, die auf den präoperativen Tumormarkerserumkonzentrationen basieren. Darüber hinaus wurden Art und Dauer der Behandlung sowie das Follow-up analysiert. Die Studie steht im Einklang mit der Deklaration von Helsinki und wurde von der lokalen Ethikkommission genehmigt (20-1493).

### Statistische Auswertung

Statistische Analysen wurden mit IBM SPSS Statistics for Windows, Version 23.0 (Armonk, NY) durchgeführt. Kontinuierliche Variablen werden als Median (Interquartilsabstand; 25. bis 75. Perzentil) dargestellt, kategorische Variablen werden als *n* (%) angegeben. Das rezidivfreie Überleben und das Gesamtüberleben wurden mit der Kaplan-Maier-Methode geschätzt. *P*-Werte < 0,05 wurden als statistisch signifikant angesehen.

## Ergebnisse

### Patientencharakteristika

Zu Beginn erfolgte die Analyse der Patientencharakteristika der 135 inkludierten Patienten mit einem metastasierten testikulären Keimzelltumor (Tab. 2). Unter Verwendung der Tumormarkerserumkonzentrationen vor Chemotherapie wurden 63 % (85/135) aller Patienten in die gute, 18 % (24/135) in die intermediäre und 19 % (26/135) in die schlechte Prognosegruppe eingeteilt (Tab. 2).

**Table 2:** 

Patientencharakteristik	Total (*n* = 135)
*Alter (Jahre)*	33 (26–41)
*IGCCCG-Klassifikation*	
Gut	63 % (85/135)
Intermediär	18 % (24/135)
Schlecht	19 % (26/135)
*Histologie*	
Seminom	30 % (41/135)
Nichtseminom	70 % (94/135)
*Lokalisation des Primärtumors*	
Testikulär	90 % (121/135)
Retroperitoneal	9 % (12/135)
Mediastinal	1 % (2/135)
*Metastasenlokalisation*^a^	
Retroperitoneale Lymphknoten	99 % (133/135)
Extraperitoneale Lymphknoten	8 % (11/135)
Pulmonal	27 % (37/135)
Viszeral	16 % (21/135)
– Leber	67 % (14/21)
– Knochen	33 % (7/21)
– Hirn	10 % (2/21)
– Andere	14 % (3/21)
*Tumormarker vor Ablatio testis*	
AFP (kU/l)	5 (2,6–75)
HCG (U/l)	21 (2–882)
LDH (U/l)	312 (200–522)
*Tumormarker vor Chemotherapie*	
AFP (kU/l)	4 (2–51)
HCG (U/l)	11,9 (0,6–555,1)
LDH (U/l)	260 (200–621)

Kontinuierliche Variablen sind angegeben als Median und Interquartilsabstand (IQR), kategoriale Variablen sind angegeben als % und Anzahl

*IGCCCG* International Germ Cell Cancer Cooperative Group, *AFP* Alpha-Fetoprotein, *HCG* humanes Choriogonadotropin, *LDH* Laktatdehydrogenase

^a^Mehrfachnennungen möglich

### Fehlklassifikation des S-Stadiums

Zunächst erfolgte eine Analyse, ob die Verwendung von Tumormarkerserumkonzentrationen vor der Ablatio testis im Vergleich zu denen vor Chemotherapie einen Einfluss auf die Bestimmung S‑Stadiums der Patienten hat. Es zeigte sich hier ein verändertes S‑Stadium bei 19 % (25/135) aller Patienten, wenn die Tumormarkerserumkonzentrationen vor der Ablatio testis im Vergleich zu denen vor Chemotherapie verwendet wurden. Hierbei kam es zu einem „up-staging“ bei 68 % (17/25) aller Patienten mit einem veränderten S‑Stadium, d. h. zu einer Eingruppierung in ein zu hohes S‑Stadium, da die Tumormarkerserumkonzentrationen nach der Ablatio testis bis zum Beginn der Chemotherapie abfielen (Tab. 3). Bei 32 % (8/25) aller Patienten mit einem veränderten S‑Stadium kam es zu einem „down-staging“, d. h. zu einer Eingruppierung in ein zu geringes S‑Stadium (Tab. 4), da die Tumormarkerserumkonzentrationen nach der Ablatio testis wieder anstiegen. Somit zeigte sich bei der Verwendung der Tumormarkerserumkonzentrationen vor der Ablatio testis im Vergleich zu denen vor Chemotherapie ein „up-staging“ des S‑Stadiums bei 13 % (17/135) und ein „down-staging“ des S‑Stadiums bei 6 % (8/135) der gesamten Patientenkohorte (Tab. 3 und 4).

**Table 3:** 

Nr	S‑Stadium inkorrekt	S‑Stadium korrekt	AFP Ablatio	AFP Chemo	HCG Ablatio	HCG Chemo	LDH Ablatio	LDH Chemo	TM	IGCCCG inkorrekt	IGCCCG korrekt	Fälschliche Therapieänderung
1	S2	S1	2.034	360	36.329	3,2	239	168	AFP	2	1	4 × statt 3 × PEB
2	S2	S1	1.743	51	21	131,5	137	176	AFP	2	1	4 × statt 3 × PEB
3	S3	S1	34.954	662	0,3	0,2	133	239	AFP	3	1	4 × statt 3 × PEB
4	S2	S1	3	4	12.025	1.258	314	327	HCG	2	1	4 × statt 3 × PEB
5	S2	S1	3	3	1,8	0,1	388	263	LDH	2	1	4 × statt 3 × PEB
6	S2	S1	43	11	0,6	0,6	929	177	LDH	2	1	4 × statt 3 × PEB
7	S2	S1	20	3	648,9	369,6	480	235	LDH	2	1	4 × statt 3 × PEB
8	S2	S1	24	18	32	10,7	397	260	LDH	2	1	4 × statt 3 × PEB
9	S3	S2	65	175	102,9	2.827	3.280	1.047	LDH	–	3	Keine Änderung (M1b, poor → intermediate)
10	S3	S2	0,1	0	104	139	1.200	944	LDH	–	2	Keine Änderung (poor → intermediate)
11	S3	S1	3	3	18.8000	2.400	178	200	HCG	–	3	Keine Änderung (M1b)
12	S2	S1	3	3	5.435	2.087	298	290	HCG	–	1	Keine Änderung (Seminom)
13	S3	S2	1,5	2	355	83	3.900	490	LDH	–	1	Keine Änderung (Seminom)
14	S2	S0	1	2	0,1	0,1	414	240	LDH	–	1	Keine Änderung (Seminom)
15	S2	S0	2	2	236	0,1	1.136	215	LDH	–	1	Keine Änderung (Seminom)
16	S2	S0	1	1	1	1	487	178	LDH	–	1	Keine Änderung (Seminom)
17	S2	S1	11,2	8	4,8	4,9	423	246	LDH	–	1	Keine Änderung (Seminom)

*AFP* Alpha-Fetoprotein, *HCG* humanes Choriogonadotropin, *LDH* Laktatdehydrogenase, *TM* Tumormarker, *IGCCCG* International Germ Cell Cancer Cooperative Group, dabei 1 = gute Prognosegruppe, 2 = intermediäre Prognosegruppe, 3 = schlechte Prognosegruppe

**Table 4:** 

Nr	S‑Stadium inkorrekt	S‑Stadium korrekt	AFP Ablatio	AFP Chemo	HCG Ablatio	HCG Chemo	LDH Ablatio	LDH Chemo	TM	IGCCCG inkorrekt	IGCCCG korrekt	Fälschliche Therapieänderung
1	S1	S2	120	1.286	509	305	278	230	AFP	1	2	3 × statt 4 × PEB
2	S1	S2	3	3	1.807	1.709	338	881	LDH	1	2	3 × statt 4 × PEB
3	S1	S2	3,4	4	9,8	7,2	187	704	LDH	1	2	3 × statt 4 × PEB
4	S2	S3	1.996	31.930	5.519	187.340	442	1.075	AFP, HCG, LDH	–	3	Keine Änderung (intermediate → poor)
5	S2	S3	7,3	7,8	38.000	52.013	280	280	HCG	–	3	Keine Änderung (intermediate → poor)
6	S1	S2	100,8	2.068	0,5	1	290	288	AFP	–	3	Keine Änderung (M1b)
7	S0	S2	2,2	5	0,1	5	231	550	LDH	–	1	Keine Änderung (Seminom)
8	S0	S2	3	1	0,2	398	196	1.240	LDH	–	1	Keine Änderung (Seminom)

*AFP* Alpha-Fetoprotein, *HCG* humanes Choriogonadotropin, *LDH* Laktatdehydrogenase, *TM* Tumormarker, *IGCCCG International Germ Cell Cancer Cooperative Group*, dabei 1 = gute Prognosegruppe, 2 = intermediäre Prognosegruppe, 3 = schlechte Prognosegruppe

### Fehlklassifikation der Prognosegruppe entsprechend der IGCCCG-Klassifikation

Da das IGCCCCG-Klassifikationssystem nicht nur auf den Tumormarkerserumkonzentrationen, sondern auch auf weiteren Parametern wie dem histologischen Subtyp, der Lokalisation des Primärtumors und der Metastasen beruht, erfolgte nachfolgend die Miteinbeziehung dieser Parameter in die Reklassifikation bei der Verwendung von Tumormarkerserumkonzentrationen vor der Ablatio testis im Vergleich zu denen vor Chemotherapie. Es zeigte sich, dass 9 Patienten aufgrund des histologischen Subtyps Seminom und 3 Patienten aufgrund des gleichzeitigen Vorliegens von viszeralen Metastasen (M1b) keine Änderung der IGCCCG-Klassifikation erfahren hätten, obwohl ein verändertes S‑Stadium vorgelegen hätte. 3 Patienten erfuhren eine Regruppierung von einer intermediären in eine schlechte Prognosegruppe bzw. schlechte intermediäre, die jedoch keine veränderte Chemotherapieanzahl nach sich zieht (Tab. 3 und 4).

Unter Berücksichtigung aller relevanten Parameter zeigte sich, dass es bei 8 % (11/135) aller Patienten zu einer veränderten Eingruppierung entsprechend der IGCCCG-Klassifikation kam, wenn diese auf den Tumormarkerserumkonzentrationen vor der Ablatio testis im Vergleich zu denen vor Chemotherapie würde (Tab. 3 und 4). Hierbei würde es zu einem „up-staging“ der Prognosegruppen bei 73 % (8/11) dieser fehlklassifizierten Patienten kommen, d. h. zu einer Eingruppierung in eine zu hohe Prognosegruppe, was 6 % (8/135) der gesamten Patientenkohorte darstellt (Tab. 3). Von diesen wären 7 Patienten in die intermediäre statt in eine gute Prognosegruppe eingeteilt worden und 1 Patient in eine schlechte statt in eine gute Prognosegruppe (Tab. 3). Als Folge dieser Fehlklassifikation würden diese Patienten eine zu intensive Therapie, nämlich 4 Zyklen PEB-Chemotherapie (Cisplatin, Etoposid, Bleomycin) anstelle von 3 Zyklen PEB-Chemotherapie erhalten (Tab. 3).

Bei 27 % (3/11) aller Patienten in dieser Untersuchungsgruppe würde es zu einem „down-staging“ kommen, d. h. Eingruppierung in eine zu geringe Prognosegruppe, die 2 % (3/135) der gesamten Patientenkohorte ausmachen (Tab. 4). Alle 3 Patienten wären in eine gute statt in eine intermediäre Prognosegruppe eingeteilt worden und hätten aufgrund dessen eine Untertherapie erhalten mit 3 Zyklen PEB-Chemotherapie anstelle von 4 Zyklen PEB-Chemotherapie erhalten (Tab. 4). Bei einem Patienten basierte die Fehlklassifikation ausschließlich auf dem Tumormarker LDH, sodass dieser Patient in die gute und nicht in die intermediäre Prognosegruppe klassifiziert wurde und somit 3 Zyklen Chemotherapie erhielt.

Eine Fehlklassifikation aufgrund einer fälschlichen IGCCCG-Klassifikation hatte in unserer Patientenkohorte keinen Einfluss auf der rezidivfreie Überleben (*p* = 0,245) sowie Gesamtüberleben (*p* = 0,992).

## Diskussion

Unsere Studie zeigte, dass die Verwendung von Tumormarkerserumkonzentrationen vor der Ablatio testis im Vergleich zu denen vor Chemotherapie in Berechnung der IGCCCG-Prognosegruppe zu einer signifikanten Fehlklassifikation führen kann, am häufigsten zu einem „up-staging“, seltener zu einem „down-staging“. Hintergrund ist, dass sowohl der tumortragende Hoden als auch Metastasen eine relevante Quelle der Tumormarkerproduktion darstellen, sodass die Metastasenlast erst nach Entfernung des Hodens, also vor Beginn der Chemotherapie, adäquat eingeschätzt werden kann [1].

Eine vorherige retrospektive Studie mit 83 Patienten zeigte eine inkorrekte Klassifikation unter Verwendung der präoperativen Tumormarkern bei 16 % aller Patienten [5]. Hier zeigte sich ein „up-staging“ von einem guten zu einem intermediären Risiko bei 10 %, sowie von einem intermediären zu einem schlechten Risiko bei 5 % aller Patienten. 1 Patient (1 %) wurde bei Verwendung der präoperativen Tumormarkern in eine zu niedrige Prognosegruppe eingestuft („down-staging“, [5]). In unserer Studie zeigte sich eine geringere Anzahl im Hinblick auf eine Änderung der IGCCCG-Prognosegruppen (6 % „up-staging“, 2 % „down-staging“*) *mit signifikanter Therapieänderung, jedoch vergleichbare Zahlen im Hinblick auf eine Änderung des S‑Stadiums (13 % „up-staging“; 6 % „down-staging“). Die Unterschiede erklären sich daraus, dass für die IGCCCG-Prognosegruppenzuordnung weitere Parameter wie der histologische Subtyp, die Lokalisation des Primärtumors und der Metastasen beachtet werden müssen [1]. In der oben erwähnten Publikation nicht beschrieben wurde jedoch, ob diese zusätzlichen relevanten Parameter in die Berechnungen inkludiert wurden, und ob die Patienten schlussendlich eine Therapieänderung erfahren hätten, was z. B. bei einer Prognoseänderung von intermediär nach schlecht nicht das Fall gewesen wäre [1]. Somit soll im Rahmen dessen erneut betont werden, dass entsprechend der aktuellen EAU-Leitlinien alle notwendigen Parameter für eine Einordnung in eine Prognosegruppe verwendet werden sollten. Von großer Bedeutung ist hier, dass die Tumormarkerserumkonzentrationen nur bei Nichtseminomen eine Rolle für die IGCCCG-Klassifikation spielen (Tab. 1; [1, 8]).

Weiterhin stellt sich die Frage, ob ein „up-grading“ der Prognosegruppe ausschließlich aufgrund des Tumormarkerserumkonzentration von LDH erfolgen sollte, da LDH als Hämolyseparameter nur einen unspezifischen Tumormarker darstellt [1]. Aufgrund dessen wurde ein Patient unserer Kohorte mit einer alleinigen LDH-Erhöhung auf 704 U/l vor Chemotherapie bei normwertigen AFP und HCG in die gute und nicht in die intermediäre Prognosegruppe eingeordnet und mit 3 Zyklen PEB-Chemotherapie behandelt. Mit der Bedeutung der drei Hodentumormarker für die Diagnostik und Therapie beschäftigt sich aus diesem Grund aktuell die deutsche Hodentumorgruppe. Sie konnte zeigen, dass die Tumormarkerserumkonzentrationen von AFP und LDH bei Patienten mit intermediärer Prognose als Prognoseparameter für das Gesamtüberleben dienen können und LDH auch v. a. bei HCG-positiven Tumoren eine große Rolle zu spielen scheint [13, 14]. Hier bedarf es jedoch sicherlich noch weiterer Analysen, die möglicherweise auch zu einer Neuaufstellung der IGCCCG-Klassifikation, welche erstmals im Jahr 1997 beschrieben wurde, führen könnte [8]. Hier sollte sicherlich auch der neue, vielversprechende Marker miR-371a-3p inkludiert werden [12].

Eine fehlerhafte IGCCCG-Gruppierung ist von enormer klinischer Relevanz, da diese entsprechend aktueller Studien zu einer signifikanten, nicht-leitliniengerechten Über- bzw. Untertherapie der Patienten und somit unnötigen Folgemaßnahmen führen kann. Vor allem beim testikulären Keimzelltumor, dem häufigsten Tumor des jungen Mannes, ist aufgrund des jungen Durchschnittsalters eine Übertherapie mit dem Risiko von Zweitmalignomen und chronischen Langzeitschäden wie Hörverlust, Fatigue, Herzkreislauferkrankungen oder neurologischen Beeinträchtigungen assoziiert [3, 4, 6, 7, 9, 10]. Obwohl wir in der aktuellen Studie – am ehesten aufgrund der geringen Patientenzahl – keinen Einfluss einer fehlerhaften Therapie auf Rezidiventstehung und Gesamtüberleben nachweisen konnten, zeigte unsere Arbeitsgruppe in einer früheren Studie, dass eine nicht-leitliniengerechte Therapie zu einem reduzierten rezidivfreie Überleben führen kann [11, 16]. Zur weiteren Evaluation dieser relevanten Thematik haben wir bereits eine multizentrische Studie mit größerem Patientenkollektiv initiiert.

Zusammenfassend sollten für eine leitliniengerechte IGCCCG-Klassifikation die Tumormarkerserumkonzentrationen vor der Chemotherapie verwendet werden. Nur so können Fehlklassifizierungen vermieden werden, die zu einer Über- oder Untertherapie führen können.

## Fazit für die Praxis

- Die Verwendung von Tumormarkerserumkonzentrationen zur Berechnung der IGCCCG-Klassifikation (International Germ Cell Cancer Cooperative Group) vor der Ablatio testis im Vergleich zu denen vor Chemotherapie kann zu einer signifikanten Fehlklassifikation führen.
- Fehlklassifikationen können in einer relevanten Über- oder Untertherapie resultieren.
- Für eine leitlinienkonforme Klassifikation der Patienten sollten somit die Tumormarkerserumkonzentrationen vor der Chemotherapie verwendet werden.
- Weitere Parameter für die IGCCCG-Prognosegruppenzuordnung sind der histologische Subtyp, die Lokalisation des Primärtumors und der Metastasen.
